# m6A methylated EphA2 and VEGFA through IGF2BP2/3 regulation promotes vasculogenic mimicry in colorectal cancer via PI3K/AKT and ERK1/2 signaling

**DOI:** 10.1038/s41419-022-04950-2

**Published:** 2022-05-21

**Authors:** Xin Liu, Hongjuan He, Fengwei Zhang, Xin Hu, Fanqi Bi, Kai Li, Haoran Yu, Yue Zhao, Xiangqi Teng, Jiaqi Li, Lihong Wang, Yan Zhang, Qiong Wu

**Affiliations:** 1 School of Life Science and Technology, State Key Laboratory of Urban Water Resource and Environment, Harbin Institute of Technology, Harbin, 150001 Heilongjiang China; 2 School of Life Science and Technology, Computational Biology Research Center, Harbin Institute of Technology, Harbin, 150001 Heilongjiang China; 3 Department of Urology, Xiang’an Hospital of Xiamen University, Xiamen, 361000 Fu Jian China

**Keywords:** Cell growth, Tumour angiogenesis

## Abstract

Exploring the epigenetic regulation mechanism of colorectal cancer (CRC) from the perspective of N6-methyladenosine (m6A) modification may provide a new target for tumor therapy. Analysis using high-throughput RNA-seq profile from TCGA found that the gene expression of Methyltransferase-like 3 (METTL3) was significantly upregulated among 20 m6A binding proteins in CRC, which was also validated in CRC cancer tissues and cell lines. Moreover, transcriptome sequencing in METTL3 knockdown cells using CRISPR/Cas9 editing suggested that EphA2 and VEGFA were differential expression, which were enriched in the vasculature development, PI3K/AKT and ERK1/2 signal pathway through the functional enrichment analysis. The results in vitro revealed that METTL3 as the m6A “writers” participates the methylation of EphA2 and VEGFA, which were recognized by the m6A “readers”, insulin-like growth factor 2 mRNA binding protein 2/3 (IGF2BP2/3), to prevent their mRNA degradation. In addition, EphA2 and VEGFA targeted by METTL3 via different IGF2BP-dependent mechanisms were found to promote vasculogenic mimicry (VM) formation via PI3K/AKT/mTOR and ERK1/2 signaling in CRC. The study suggests that intervention with m6A-binding proteins (METTL3 and IGF2BP2/3) may provide a potential diagnostic or prognostic target of VM-based anti-metastasis drugs for CRC.

## Introduction

Colorectal cancer (CRC) is among the 10 most common cancers worldwide, ranking third for incidence, but second for mortality, and the incidence rate is rising worldwide [1]. The identification of prognostic and predictive biomarkers for early diagnosis, prevention, and targeted therapy is a major challenge for CRC researchers. Although many studies have clarified the biological characteristics of CRC, morbidity and mortality remain high among patients with this type of cancer. Therefore, it is essential to understand how CRC develops and identify therapeutic targets to improve patient prognosis.

To meet the demands caused by their growth and development, tumors need to form new blood vessels to transport nutrients and oxygen [2, 3]. In addition to traditional tumor angiogenesis, vasculogenic mimicry (VM), which is independent of endothelial cells, was first described as a novel process by Maniotis et al. in 1999 [4]. VM is a new tumor microcirculation model; the blood vessels formed in VM can transport sufficient nutrients and blood supply to support tumor growth [4]. Further, VM is closely associated with high tumor metastasis and poor patient prognosis [5], and has emerged as a promising new target for anti-tumor therapy [6]. VM has been observed in a variety of malignant tumors including hepatocellular carcinoma [7], breast cancers [8], colorectal cancer [9] and prostate cancer [10], among others. Meanwhile, some key VM-related molecules have been investigated in various aggressive malignant tumors, including hypoxia-inducible factors [11], Ephrin type-A receptor 2 (EphA2) [12, 13] vascular endothelial growth factor (VEGF, also called VEGFA) [14], and matrix metalloproteinases [15]. EphA2 belongs to erythropoietin-producing hepatocytes family and is a transmembrane tyrosine kinase growth receptor associated with the of various malignant tumors [16, 17] and involved in mediating VM formation. In gastric cancer cells, cancer‑associated fibroblasts over-expressing EphA2 promote VM formation by activating the EphA2‑PI3K pathway [12]. In human glioma, miR‑141 directly targets *EphA2* and inhibits VM by controlling EphA2 expression [13]. VEGFA plays an important role in the formation of VM; it can bind and activate its tyrosine kinase receptors, VEGFR1 and VEGFR2, to influence VM formation in different tumor cell types [18, 19].

N^6^-methyladenosine (m6A) is the RNA post-transcriptional modification that has been best studied to date, and is enriched in mRNA 3ʹ untranslated regions (UTRs) adjacent to stop codons, or near the last exon of noncoding RNA molecules, as well as around start codons in *Arabidopsis thaliana* [20–22]. In addition, m6A modification preferentially occurs in the consensus sequence, “RRACH” (where R = G or A, and H = A, C or U) [20, 22]. m6A modification is reversible and can be dynamically regulated; generation of m6A modifications is catalyzed by a methyltransferase complex composed of METTL3/METTL14/WTAP proteins, also termed “writers”. “Eraser” demethylases (e.g., FTO, ALKBH5, and ALKBH3) can remove the m6A modification, and the m6A residue can be recognized by “readers” (e.g., YTHDC1/2, YTHDF1/2/3, IGF2BP1/2/3, HNRNP, and eIF3) [23]. m6A plays important roles in mRNA stabilization, splicing, degradation, and translation efficiency via altering target gene expression [24, 25], and its function has been explored in various biological processes, including stem cell differentiation [26], embryonic development [27], DNA damage [28], and tumor progression [23], among others. As an important m6A regulator enzyme, the methyltransferase, METTL3, can promote tumor progression by regulating downstream target genes in leukemia [29], colorectal cancer [30, 31], bladder cancer [32], and gastric cancer [33]; however, the relationship between the m6A writer, METTL3, and VM formation remains unclear.

In this study, we found that METTL3 was highly expressed in CRC and that its expression levels were correlated with poor patient prognosis. RNA sequencing and functional studies suggested that METTL3 could target *EphA2* and *VEGFA*, and that the resulting m6A modification can inhibit *EphA2* and *VEGFA* mRNA degradation via a novel m(6)A-IGF2BP-dependent mechanism. Moreover, METTL3 knockdown inhibited CRC proliferation, migration, invasion, and VM formation in vitro, and this could be rescued by overexpression of EphA2 and VEGFA. Furthermore, METTL3-mediated modification of *EphA2* and *VEGFA* influenced VM formation via activating both the PI3K/AKT and ERK1/2 signaling pathways in vitro and in vivo. Our findings demonstrate the critical role of m6A modification by METTL3 in VM formation, and highlight METTL3 as a potential therapeutic target, via impairing VM formation, in anti-CRC metastasis strategies.

## Results

### METTL3 is highly expressed in CRC and associated with clinical features

**Fig. 1:**
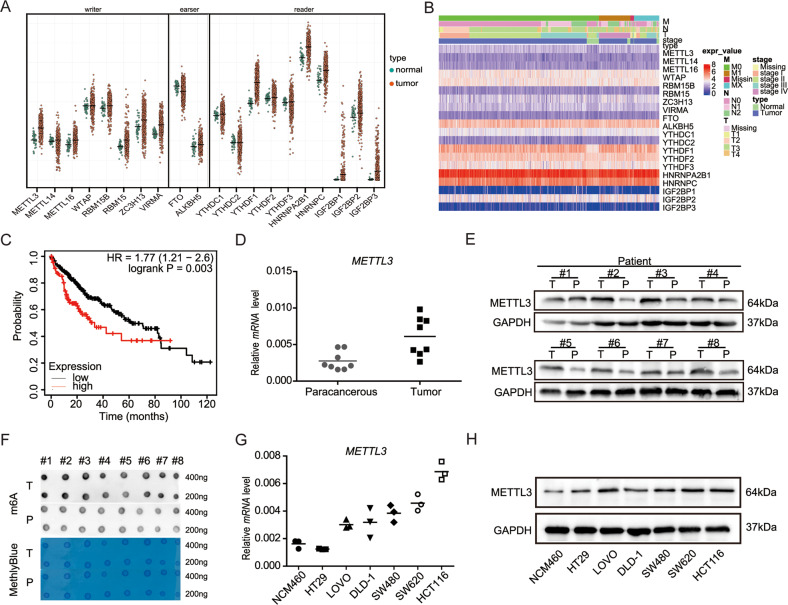
METTL3 is highly expressed in CRC and associated with clinical features. **A**, **B** Heat map profile and histogram showing the expression of m6A modification related genes in the COAD TCGA database. **C** Kaplan-Meier analysis of the association of METTL3 expression with overall survival in COAD patients. **D**, **E** qRT-PCR and western blot analysis of METTL3 expression in CRC tissues from eight patients. **F** m6A dot blot assay of tissue samples (cancer and paracancer) from eight patients with CRC. MB, methylene blue staining (as loading control). **G**, **H** Real-time PCR and immunoblotting analyses of METTL3 expression in normal colonic epithelial and CRC cell lines.

The function of m6A modification is determined by writers, erasers and readers, and the abnormality of its regulation mechanism is related to cancer [23, 26, 27]. In order to reveal the role of m6A modification in CRC, the gene expressions of m6A binding proteins were compared by RNA-seq in TCGA. The results showed that four writers (METTL3, METTL16, RBM15, and VIRMA) and six readers (YTHDF1, YTHDF2, HNRNPA2B1, HNRNPC, and IGF2BP2/3) were significantly upregulated in CRC tumors compared to adjacent normal tissues. However, the gene expression of eraser, FTO, was downregulated, while that of other m6A binding proteins did not differ significantly (Fig. 1A). Figure 1B showed METTL3 had differential expression in different stages. Additionally, Kaplan-Meier survival analysis revealed that CRC patients with high METTL3 expression exhibited a poor overall survival compared with patients with low METTL3 levels (Fig. 1C).

Next, we evaluated METTL3 expression patterns in 8 paired CRC samples and 6 CRC cell lines using qRT-PCR and western blotting. The results indicated that METTL3 expression was notably higher in CRC patient tissues than in adjacent normal tissues (Fig. 1D, E). Furthermore, the results of an m6A dot blot assay indicated that global m6A levels were clearly increased, alongside the high expression of METTL3, in CRC tissues, suggesting that m6A levels are increased in human CRC. Compared with expression in the normal colonic epithelial cell line, NCM460, METTL3 levels were also significantly upregulated in six CRC cell lines (HT29, LoVo, DLD-1, SW480, SW620, and HCT116) at both at mRNA and protein levels, consistent with the results from CRC tissues (Fig. 1G, H). HCT116 cells had relatively high METTL3 expression levels, and were therefore selected for use in further research.

### Transcriptome-sequencing identified *EphA2* and *VEGFA* as direct targets of METTL3 that influence vasculature development

**Fig. 2:**
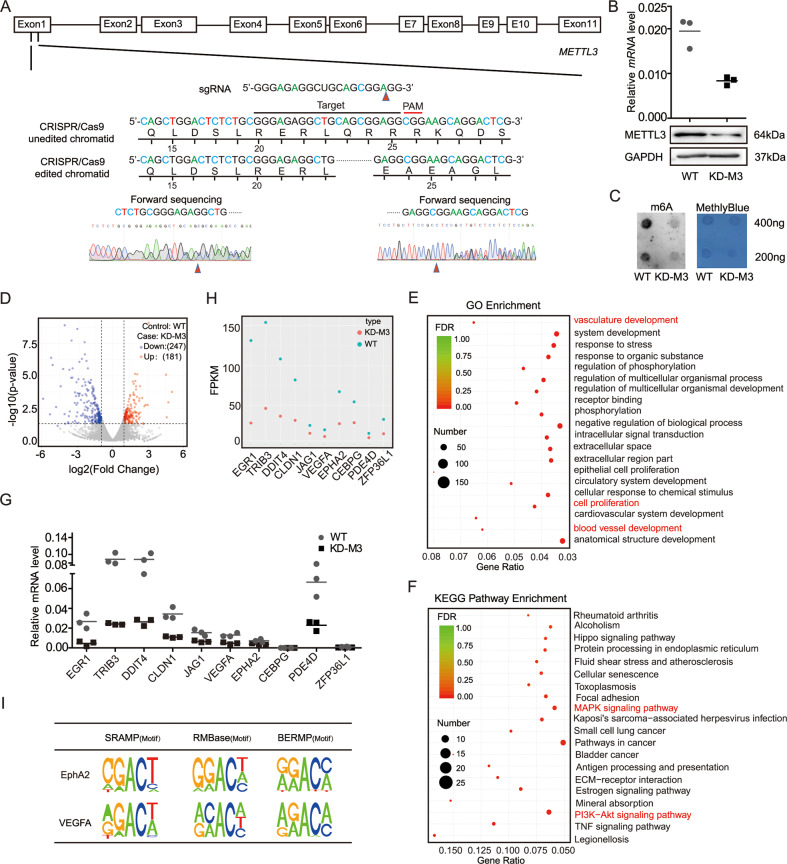
Transcriptome-sequencing identified *EphA2* and *VEGFA* as direct targets of METTL3 that influence vasculature development. **A** Schematic of CRISPR/Cas9-mediated deletion of METTL3 and identification of METTL3 deletion clones by sequencing. **B** METTL3 expression levels were significantly decreased in METTL3-knockdown cells, as demonstrated by qRT-PCR, western blotting, and (**C**) m6A dot blot analysis. **D** RNA-Seq identified differentially expressed genes in METTL3 stable knockdown cells compared with their corresponding controls. **E**, **F** GO enrichment and KEGG analysis of differentially expressed genes. **G**, **H** Scatter diagram and qRT-PCR analysis of the expression of the top ten most significantly down-regulated genes. **I** Identification of the METTL3 consensus motif (GGAC) in the target *EphA2* and *VEGFA* mRNAs using SRAMP/RMBase2.0/BERMP website tools. The data in (**B**) and (**H**) were normalized to *GAPDH* expression levels. *p* values were calculated using *t* test from three independent experiments. ***p < 0.001.

The most important function of the METTL3 binding protein in cancer is the identification of the target genes. In this study, the sgRNA sequence targeting *METTL3* exon 1 is shown in Fig. 2A. We firstly used the CRISPR/Cas9 editing system to generate a stable METTL3-knockdown HCT116 cell line. The optimal single guide RNA (sgRNA) was selected as the target sequence from 65 sgRNA according to the Crispr Can (https://www.Crispr.org) and Crispr Direct (http://Crispr.dbcls.jp) website (see methods and materials). Knockdown of METTL3 was analyzed by sequencing, and knockdown efficiency further validated by qRT-PCR and western blotting. The results indicated that METTL3 expression was effectively knocked down (Fig. 2A, B). Further, dot blot analysis showed that METTL3 knockdown reduced the global m6A modification level in HCT116 cells (Fig. 2C).

Next, to investigate the target genes that can be regulated by METTL3 via the m6A pathway, we performed RNA-seq using wild-type and stable METTL3-knockdown HCT-116 cells. RNA-seq analysis identified 247 significantly down-regulated and 181 up-regulated genes (Fig. 2D). These genes were further analyzed by GO and KEGG pathway enrichment analyses. GO analysis of biological processes suggested that METTL3 may affect tumor-related biological processes, such as vasculature development and cell proliferation, among others. Interestingly, we found that the DEGs were most strongly enriched in the MAPK and PI3K-AKT signaling pathways (Fig. 2E, F).

Based on fragments per kilobase of transcript per million mapped reads data we identified 10 candidate genes significantly down-regulated in stable METTL3-knockdown cells for further confirmation. The results of qRT-PCR analysis indicated that the expression levels of *EGR1*, *TRIB3*, *DDIT4*, *CLDN1*, *JAG1*, *PDE4D*, as well as those of *VEGFA* and *EphA2*, were dramatically decreased in stable METTL3-knockdown cells relative to wild-type cells (Fig. 2G, H). To verify the role of m6A modification in METTL3-mediated gene regulation, m6A sites were predicted using the SRAMP database (http://www.cuilab.cn/sramp), RMBase2.0, and BERMP. The score of *EphA2* and *VEGFA* were displayed significantly with “RRACH” motif among the 10 DEGs, meanwhile, the 3ʹUTR regions adjacent to the stop codon of the *EphA2* and *VEGFA* mRNAs contained the highest confidence level of m6A modification (Fig. 2I and Table S2). Taken together, the above results suggested that the EphA2 / VEGFA may be regulated by METTL3.

### Identified EphA2 and VEGFA as targeted regulation gene by METTL3-mediated m6A modification

**Fig. 3:**
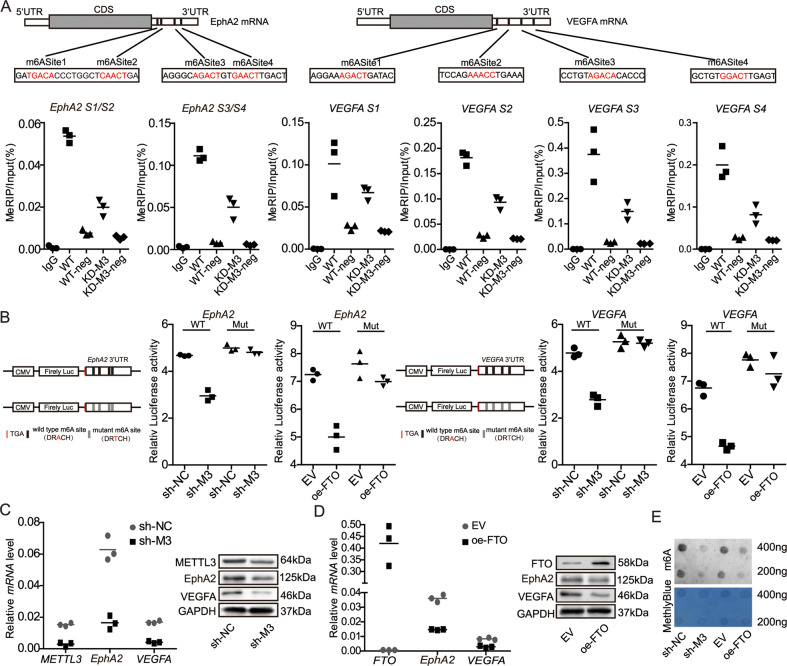
Identified EphA2 and VEGFA as targeted regulation gene by METTL3-mediated m6A modification. **A** Clear m6A modification of *EphA2* and *VEGFA* confirmed by MeRIP-qPCR, and knockdown of the m6A writer, METTL3, repressed this m6A modification. **B** Relative luciferase activity of pMIR-REPORT-EphA2 and pMIR-REPORT-VEGFA with either wild-type or mutant (A-to-T mutation) m6A sites after co-transfection with shNC/METTL3 or pcDNA3.1/pcDNA3.1-FTO into HEK-293T cells. Firefly luciferase activity was measured and normalized to that of Renilla luciferase. **C**, **D** Expression levels of EphA2 and VEGFA detected by qRT-PCR and western blotting after treatment of cells with shMETTL3 and FTO overexpression. **E** The m6A level in HCT116 cells treated with shMETTL3 and FTO overexpression. Data in (**A**), **C**, **D** were normalized to GAPDH levels. *p* values were calculated using the *t* test from three independent experiments. ***p < 0.001. Antibodies against GAPDH and IgG were used as negative controls. Relative EphA2 and VEGFA enrichment in the RIP assay was normalized by input levels.

To further investigate whether METTL3 targets *EphA2* and *VEGFA* mRNA via m6A modification, we conducted MeRIP-qPCR to investigate the enrichment of m6A in *EphA2* and *VEGFA* (Fig. S1). Our results showed that m6A abundance was markedly decreased in *EphA2* and *VEGFA* mRNA molecules after METTL3 knockdown (Fig. 3A). More importantly, to assess the effects of target mRNA m6A modification on gene regulation, we conducted luciferase reporter assays using constructs containing either wild-type or mutant *EphA2* and *VEGFA* m6A sites to assess the effects of m6A modification on *EphA2* and *VEGFA* expression. In the mutated *EphA2* and *VEGFA* constructs, the adenosine bases (A) in m6A consensus sequences (RRACH) were substituted with thymine (T), to abolish the m6A modification. As expected, luciferase reporter assays revealed that the m6A modification of wild-type, but not mutated, *EphA2* and *VEGFA* was clearly decreased in cells with METTL3 knockdown (sh-M3) or overexpressing the m6A demethylase, FTO (ov-FTO) (Fig. 3B). In addition, qRT-PCR and western blot analysis confirmed that METTL3 inhibition or FTO overexpression decreased EphA2 and VEGFA expression levels in HCT116 cells (Fig. 3C, D). Meanwhile, reduced METTL3 or overexpression of FTO resulted in decreased m6A levels (Fig. 3E). These data demonstrate that *EphA2* and *VEGFA* are targets of METTL3 and regulated by METTL3 in an m6A-dependent manner.

### METTL3 knockdown enhances *EphA2* and *VEGFA* mRNA stability via an m6A-IGF2BP2/3-dependent pathway

**Fig. 4:**
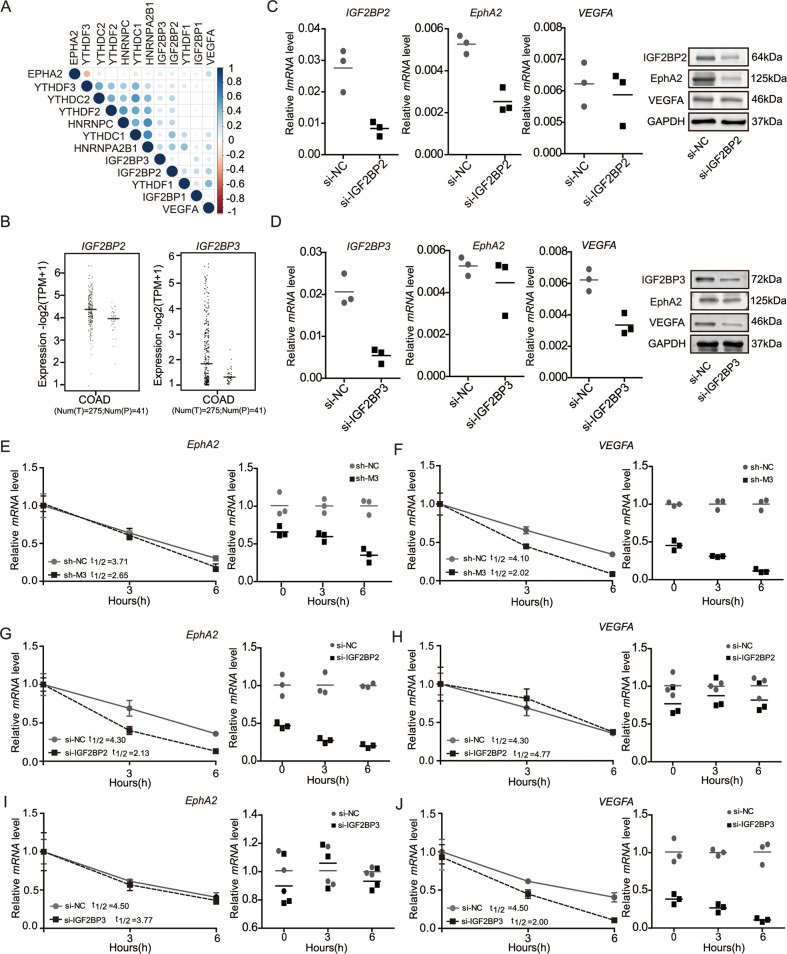
METTL3 knockdown enhances *EphA2* and *VEGFA* mRNA stability via an m6A-IGF2BP2/3-dependent pathway. **A** The association of *EphA2/VEGFA* and m6A readers in colon cancer analyzed based on TCGA database. **B** The mRNA expression of *IGF2BP2/3* in colon cancer analyzed using the GEPIA website tool based on TCGA. **C**, **D** Real-time PCR and western blot assay of EphA2 and VEGFA expression in HCT116 cells after depletion of m6A readers (IGF2BP2/3). **E**, **F** The decay rate of mRNA and qPCR analysis of *EphA2* and *VEGFA* at the indicated times after actinomycin D (5 μg/ml) treatment in HCT116 cells after METTL3 inhibition. **G**–**J** The decay rate of mRNA and qPCR analysis of *EphA2* and *VEGFA* at the indicated times after actinomycin D (5 μg/ml) treatment in HCT116 cells after IGF2BP2/3 inhibition. *p* values were calculated using the *t* test from three independent experiments. *p < 0.05, **p < 0.01 (Student’s *t* test).

Previous studies identified two major families of m6A “readers”, the YTH and the IGF2BP families, that play specific roles in controlling the fate of methylated mRNA. IGF2BP proteins can function by recruiting RNA stabilizers, such as HuR, to protect m6A-containing mRNA molecules from degradation [34]. To further test our hypothesis that the EphA2/VEGFA axis can be regulated by METTL3 in an m6A-dependent manner and to elucidate the specific m6A readers of *EphA2* and *VEGFA*, we first analyzed the correlation between IGF2BP proteins and EphA2/VEGFA expression using COAD analysis of TCGA data. The results showed that IGF2BP levels were positively correlated with those of *EphA2* and *VEGFA* (Fig. 4A), indicating a potential positive regulatory mechanism. Further, analysis of RNA sequencing expression data in the GEPIA on-line database (http://gepia.cancer-pku.cn) showed higher expression levels of *IGF2BP2/3* in CRC, based on TCGA data (Fig. 4B). Therefore, we further explored the effects of IGF2BP2 and IGF2BP3 on *EphA2* and *VEGFA* mRNA stability. Two specific siRNAs targeting IGF2BP2/3 were used to silence their expression, and knockdown efficiency confirmed by qRT-PCR and western blot. Interestingly, we found that IGF2BP2 knockdown strongly decreased expression of *EphA2*, but not *VEGFA*, while reduced IGF2BP3 expression inhibited *VEGFA* levels in HCT116 cells (Fig. 4C, D and Fig. S2). Furthermore, we assessed the RNA decay rate in HCT116 cells with METTL3 inhibition (sh-M3) and corresponding controls (sh-NC). *EphA2* and *VEGFA* mRNA levels were initially reduced, and the half-lives of these mRNA molecules were consistently markedly shortened following METTL3 knockdown in HCT116 cells (Fig. 4E, F). Interestingly, the half-life of *EphA2* mRNA was also significantly shortened after IGF2BP2 knockdown (Fig. 4G), but did not change in cells treated with IGF2BP3 siRNA (Fig. 4I). By contrast, the *VEGFA* mRNA half-life was significantly reduced after IGF2BP3 siRNA inhibition (Fig. 4H), while it remained unchanged on IGF2BP2 inhibition (Fig. 4J).

Taken together, our data suggest that methylated *EphA2* and *VEGFA* transcripts are recognized by different m6A “readers” (i.e., IGF2BPs). IGF2BP2 bound to methylated *EphA2*, while IGF2BP3 recognizes methylated *VEGFA*, to enhance *EphA2/VEGFA* mRNA stability, prevent their degradation, and increase their expression via an m6A-IGF2BP2/3-dependent mechanism.

### METTL3 modification of EphA2/VEGFA promotes VM in CRC cells via PI3K/AKT and ERK1/2 signaling in vitro and in vivo

**Fig. 5:**
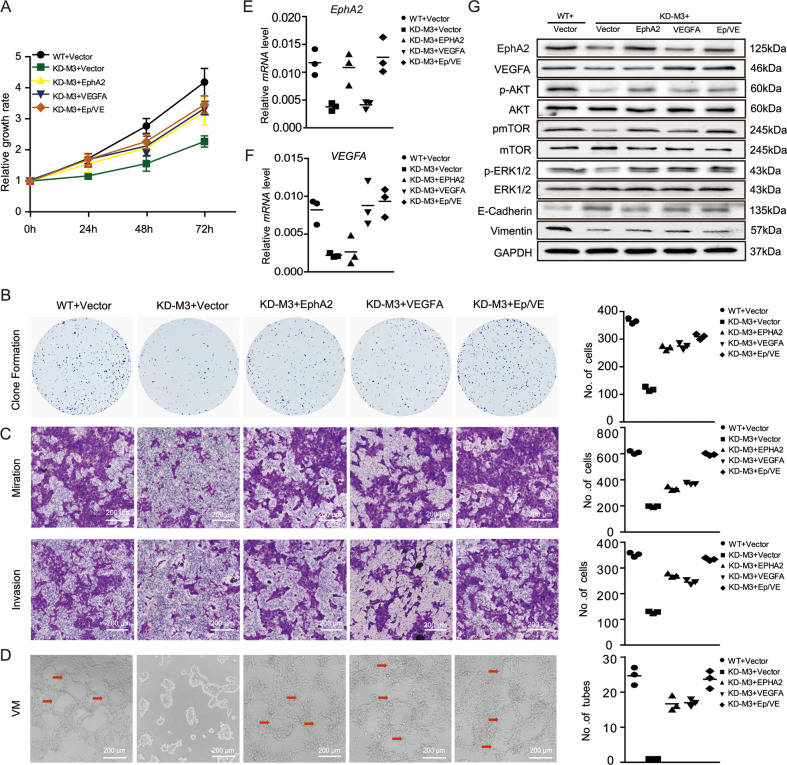
METTL3 modification of EphA2/VEGFA promotes VM in CRC cells via PI3K/AKT and ERK1/2 signaling in vitro and in vivo. **A** Cell proliferation was measured using an MTT assay after METTL3 knockdown and EphA2/VEGFA overexpression. **B** Migration and invasion efficiency elucidated by transwell assay. **C** Summary graphs showing the numbers of colonies counted. **D** Representative images of tube formation by HCT116 cells and METTL3-deletion cells overexpressing EphA2/VEGFA after 48 h. Scale bars, 200 µm. **E**–**G** EphA2 expression detected by qRT-PCR and western blotting after successful overexpression of EphA2 and VEGFA. **H** Effect of METTL3 deletion on the AKT/ERK1/2 signaling pathway. *p* values were calculated using the *t* test from three independent experiments. **p < 0.01, ***p < 0.001. WT wild-type cells, KD-M3 METTL3 knockdown, Vector, cells transfected with GV230 empty vector.

It is need that uncovering of the complex regulation mechanism of EphA2 and VEGFA with METTL3 modification. Based on our RNA-seq data analysis, EphA2 and VEGFA were enriched in growth and vasculature development in HCT116 cells (Fig. 2H); therefore, we evaluated the tumor‑promoting ability of METTL3 and its target genes, *EphA2* and *VEGFA*, by upregulating EphA2 and VEGFA using a GV230 overexpression vector. The results of an MTT assay showed that METTL3 silencing reduced cell proliferation, which could be rescued by overexpression of EphA2 or VEGFA. Additionally, the proliferation of METTL3 knockdown cells co-expressing EphA2 and VEGFA increased more than that in cells overexpressing EphA2 or VEGFA alone (Fig. 5A).

Furthermore, to confirm the effects of METTL3 on cell proliferation, we performed a clonogenic assay. The results suggested that colony formation efficiency was dramatically decreased in cells with METTL3 deleted and could be rescued by EphA2/VEGFA overexpression (Fig. 5B). Next, the role of METTL13 on CRC cell migration and invasion was explored by transwell migration and invasion assays. It is showed that METTL3 deletion significantly inhibited cell migration and invasion, whereas EphA2/VEGFA overexpression clearly impaired CRC cell migration and invasion, whether overexpressed individually or in combination (Fig. 5C).

VM is necessary for rapid tumor proliferation, and EphA2 and VEGFA are important molecules in this process. Thus, to investigate the role of METTL3-mediated modulation of EphA2/VEGFA in CRC VM, we analyzed VM formation in vitro using matrix gel and HCT116 cells. As shown in Fig. 5D, HCT-116 cells produced partial tubular structures of various sizes in a matrix gel; however, the tubular structures were not formed after METTL3 knockdown. Interestingly, VM formation by HCT-116 cells in vitro was clearly repaired by overexpression of EphA2 (KD-M3 + Ep) or VEGFA (KD-M3 + VE), with more tubular structures detected on co-overexpression of both EphA2 and VEGFA (KD-M3 + Ep/VE) (Fig. 5D). In addition, successful overexpression of EphA2 and VEGFA was confirmed by qRT-PCR and western blot (Fig. 5E–G). These data indicate that METTL3-derived EphA2/VEGFA activation contributes to VM formation.

As mentioned above, KEGG pathway analysis indicated that METTL3-mediated modulation of EphA2 and VEGFA affected VM formation via PI3K-AKT and MAPK ERK1/2 signaling (Fig. 2I). To confirm effects of EphA2/VEGFA in promoting VM formation and explore the underlying mechanisms, the expression of p‑AKT and p‑ERK1/2 were analyzed following METTL3 inhibition and overexpression of EphA2/VEGFA. The resulting data demonstrated that METTL3 knockdown inhibits the phosphorylation of AKT and mTOR, while overexpression of EphA2 and VEGFA partially reverses activation of the PI3K/AKT/mTOR pathway induced by METTL3 knockdown. Moreover, the same event leads to activation of the ERK1/2 pathway (Fig. 5G). Notably, expression of the epithelial marker, E-cadherin, was increased after knocking down METTL3, and then reduced by the overexpression of EphA2 or VEGFA. Further, the expression of vascular endothelial-cadherin (vimentin), a marker of VM, was also suppressed after METTL3 down-regulation and then increased when EphA2 and VEGFA was overexpressed.

**Fig. 6:**
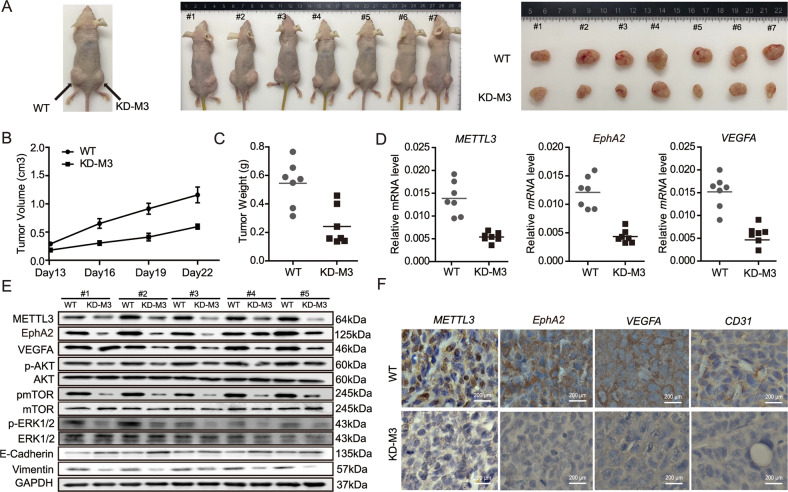
METTL3 modification of EphA2/VEGFA promotes VM in CRC cells via PI3K/AKT and ERK1/2 signaling in vitro and in vivo. **A** METTL3 deletion HCT116 cells and control cells were injected into flanks of nude mice and the representative mice were presented. **B** Tumor growth curve after the injection of METTL3-deficient HCT116 cells and control cells into nude mice (*n* = 7). **C** Tumors weight was measured. **D**, **E** The expression of EphA2/VEGFA in tumors was detected by qRT-PCR and AKT/ERK1/2 signaling pathway was detected through western blotting. **F** Representive images of IHC staining for METTL3, EphA2, VEGFA and CD31 protein on a tumor tissue. *p* values were calculated using *t* test from three independent experiments. *p < 0.05.

To further investigate the effects of METTL3 on VM formation in vivo, we established xenografts in nude mice and then injected METTL3-deleted and control HCT116 cells into the flanks of each mouse, and measured the tumor volume every 3 days after day 13. As shown in Fig. 6A–C, tumors were significantly smaller in the METTL3 knockdown group than in the control group (*p* < 0.01). Additionally, the EphA2 and VEGFA expression levels were clearly decreased in METTL3-knockdown xenografts compared with the control group (*p* < 0.001) (Fig. 6D–F). Similarly, CD31 staining revealed that VM was markedly reduced in METTL3-knockdown xenografts (Fig. 6E). Furthermore, p-AKT/p-mTOR and p-ERK1/2 expression were lower in the METTL3-knockdown group compared with the WT control group. Expression levels of vimentin were also significantly decreased in the stable METTL3-knockdown group, while those of E-cadherin were higher (Fig. 6F).

In conclusion, these data suggest that METTL3 regulates EphA2 and VEGFA via different IGF2BP-dependent mechanisms to promote VM formation via PI3K/AKT/mTOR and ERK1/2 signaling, which upregulates the expression of the VM‑related gene, vimentin, in CRC tumorigenesis.

## Discussion

**Fig. 7:**
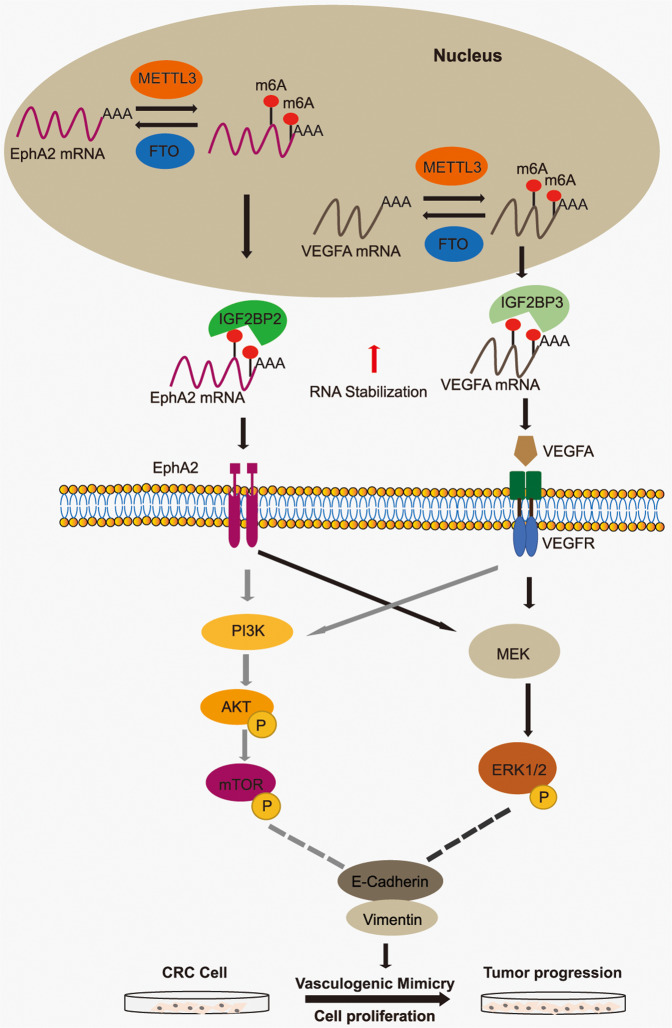
Proposed working model of the proposed mechanism in this study. Firstly, METTL3 methylation *EphA2* and *VEGFA* was subsequently recognized by the m6A “reader”, IGF2BP2/3, to maintain its mRNA stability and expression. Then the increasing expression of EphA2 and VEGFA promoted VM formation by activating both PI3K/AKT and ERK1/2 signaling pathways to facilitate CRC progression.

N6-methyladenosine methylation is a type of methylation modification on RNA molecules, which was erected by methyltransferases (“writers”) and removed by demethylases (“erasers”) and the effects on targeted mRNA depend on the functions of different m6A binding proteins (“readers”) [35]. m6A modification has been associated with tumorigenesis and progression of cancer. Research shows that METTL3 promotes cancer progression through YTHDF2 dependent posttranscriptional silencing of SOCS2 in liver cancer [36]. ALKBH5-mediated m6A-demethylation of *NANOG* mRNA is involved in hypoxia-induced breast cancer stem cell phenotype [37]. In this study, we demonstrate that the upregulated METTL3 expression in CRC results in aberrant m6A modification, and that methylation of *EphA2* and *VEGFA* by METTL3 via different IGF2BP-dependent mechanisms induces VM formation to promote CRC progression by activating both PI3K/AKT and ERK1/2 signaling (Fig. 7).

Angiogenesis and VM vessels provide growing tumors with sufficient blood perfusion and promotes cancer metastasis and progression [5]. VM was discovered play a critical role in promoting Hepatocellular carcinoma (HCC) metastasis, invasion, which led to poor prognosis [38]. By studying the migration, invasion and matrix remodeling of tumor cells to investigate the formation mechanism of VM, such as VE-cadherin [33, 39], ephrin type-A receptor 2 (EphA2) [27] and phosphoinositide 3-kinases (PI3K) [40] are involve in VM formation. Additional, one previous study demonstrated m6A “readers”, IGF2BP3, participates VM formation by sustaining VEGF expression and cell proliferation in colon cancer [41]. However, the genes that are usually recognized by a “reader” are first methylated by a “writer” [42]. METTL3 is the only catalytic subunit with methyltransferase activity in the m6A modification process, and forms the METTL3/METTL14/WTAP complex to exert RNA-modified biological activity [35]. Therefore, we detected gene expression by RNA-seq in METTL3 knocked down using CRISPR-cas9 compared with WT HCT116 cells. 428 differential expression genes were significantly enriched in the vasculature development, cell proliferation, and blood vessel development. In addition, *EphA2* and *VEGFA* among in the downregulated 10 DEGs were founded the optimal m6A motif sites by MeRIP-qPCR and Luciferase reporter assay. It is demonstrated that EphA2 and VEGFA are functionally important targets of METTL3.

Target m6A‑modified mRNAs is needed specifically recognized by m6A binding proteins (readers), which function in a variety of biological processes, including RNA splicing, transport, stability, decay, and translation [43, 44]. We speculate that one “reader” may regulate multiple genes, and a gene may be regulated by multiple “readers”. For instance, YTHDF proteins (YTHDFs) each have their own sets of target mRNAs which can be differentially regulated to different cellular signals in the cytosol; however, previous studies also have confirmed that a spatiotemporal interplay among the three YTHDFs to cooperatively control translation and decay of their common target mRNAs [44, 45]. In this study, the “readers” of *EphA2* and *VEGFA* targeted by METTL3 are IGF2BP2/3 which suppress its RNA degradation and maintain transcript stability. Thus, our data exhibits effects on mRNA distinct from previous known reading processes that different binding proteins (IGF2BPs) perform the same function of promoting VM formation by recognizing different target genes.

Vasculogenic mimicry provides growing tumors with sufficient blood perfusion and promotes cancer metastasis and progression [46]. Previous studies suggest that TGF-β [15], Hippo [47], PI3K-AKT[39, 40], and ERK1/2 [48] signaling pathways have been reported to be involved in VM among cancer. The imbalance of EphA2 and VEGFA can activate the PI3K-AKT and ERK1/2 signaling pathway which were the important intracellular signaling pathways, thereby regulating the basic functions of cells [9, 27, 31]. In this study, we revealed that the RNA-seq and the function of the target genes both point to VM which METTL3 methylated *EphA2* and *VEGFA* induces VM formation by activating both PI3K/AKT and ERK1/2 signaling which as confirmed by in vitro and in vivo.

In summary, we provide compelling in vitro and in vivo evidences demonstrating that m6A modification machinery in CRC, and reveal profound insights into the molecular mechanisms underlying tumorigenesis by revealing EphA2 and VEGFA were simultaneously recognized by two different binding proteins (IGF2BP2/IGF2BP3) and participated in the same signaling pathway (PI3K/Akt and ERK1/2) to jointly promote the formation of VM. The research may provide a potential diagnostic or prognostic target of VM-based anti-metastasis drugs for CRC.

## Materials and methods

### Tissue specimens and patient information

A total of 8 CRC and paired adjacent normal tissue specimens were randomly collected at the Fourth Hospital of Harbin Medical University (Harbin, China) with permission from the Institutional Ethics Committee for further extraction and analysis. Written informed consent was obtained from all patients. This study was approved by the Ethics Committee of Harbin Institute of Technology. In the experiment, the conscious or subconscious personal subjectivity and preference of operators and participants were excluded.

### CRC cell lines and cell culture

CRC cell lines, including HCT-116, NCM460, HT29, LOVO, DLD-1, SW480, and SW620 cells, were purchased from ATCC and cultured in DMEM (Gibco, New York, USA) supplemented with 10% fetal bovine serum (FBS; CellMax, China) and 1% penicillin-streptomycin in a 5% CO_2_ humidified incubator at 37 °C. The cell line was routinely tested to exclude mycoplasma contamination.

### RNA isolation and quantitative real-time PCR (qRT-PCR)

Total RNA was extracted from tissues and cells using RNAiso Plus (TaKaRa, China) and then cDNA was synthesized using a PrimeScript^TM^ RT reagent Kit (TaKaRa), according to the manufacturer’s instructions. mRNA expression levels were assessed by qRT-PCR using a SYBR Premix Ex Taq Kit (TaKaRa) on an ABI 7500 Real-Time PCR System, with *GAPDH* used as internal control. Primers used in qRT-PCR are listed in (Supplementary Table S1).

### Western blot analysis

Total proteins were extracted from tissue or cell specimens using RIPA lysis buffer containing PMSF and Protease Inhibitor Cocktail (APEXBIO, USA) and protein concentrations determined using a BCA kit (Beyotime, China). Then, 30 μg of whole cell or tissue lysates were resolved on 10% SDS-PAGE and transferred to PVDF membranes. After blocking with 5% skim milk /TBST, membranes were incubated with specific primary antibody at 4 °C overnight. Then, blots were washed with TBST, incubated with species-specific horseradish peroxidase (HRP) -conjugated secondary antibody (ABclonal, Cat.NO:AS014), and the signal visualized using an ECL chromogenic kit (Beyotime, China) on the Mini-REPORT Tetra Electrophoresis System. Antibodies used for western blot analysis were diluted as follows: METTL3 (Abcam, ab195352), EphA2 (CST, 6997), VEGFA (Proteintech,19003-1-AP), FTO (Abcam, ab126605), IGF2BP2 (Proteintech,11601-1-AP), IGF2BP3 (Proteintech,14642-1-AP), AKT/ p-AKT (CST, 1:1000), mTOR/p-mTOR (CST, 2983/5536), ERK1/2 and p-ERK1/2 (Proteintech, 16443-1-AP/80031-1-RR), HIF1α (CST, 36169), E-cadherin (CST, 3195), Vimentin (CST, 5741), and GAPDH (Abcam, ab181602). The original pictures of western blot are listed in the supplementary material.

### m6A dot blot assays

m6A dot blotting was performed as previously described [49]. Briefly, isolated total RNA was measured using a NanoDrop instrument, and 200–400 ng RNA was spotted onto a Hybond-N + membrane (Invitrogen, USA). Then, membranes were crosslinked using UV irradiation and incubated with m6A-specific antibody (Abcam, 1:1000 dilution) overnight at 4 °C, after blocking with 5% nonfat milk in PBST. Then, diluted HRP-conjugated secondary antibody (1:10000) was added to the membrane for 1 h at room temperature. Membranes were developed using an ECL chromogenic kit (Beyotime, China) and the signal detected using a the Mini-REPORT Tetra Electrophoresis System. Methylene blue staining was used as a loading control.

### METTL3 knockdown

To construct a METTL3 knockdown HCT116 cell line, a single guide RNA (sgRNA) for a frameshift mutation of the *METTL3* (sequence: GAGAGGCTGCAGCGGAGG) was designed by Crispr Can (https://www.Crispr.org) and Crispr Direct (http://Crispr.dbcls.jp). Annealed double-stranded DNA was inserted into the px458 vector using the BbsI (NEB, USA) site. Then the purified recombinant plasmid was transfected into HCT-116 cells using Lipofectamine 3000 (Invitrogen). After puromycin screening, cells were separated into 96-well plates by limiting dilution and collected to test the knockdown efficiency. The resulting screened, stable METTL3 knockdown cell line was used for phenotypic and tumor xenograft experiments in vivo.

For luciferase reporter assays, a short hairpin RNA plasmid targeting METTL3 purchased from Gene Pharma (Suzhou, China) was transfected into HCT116 cells to knock down METTL3 expression. For FTO overexpression, *FTO* cDNA was amplified and subcloned into the pcDNA3.1 vector. After transfection, METTL3 and FTO expression levels were detected by qPCR and western blot analyses. A GV230-EphA2 overexpression vector was purchased from Gene Chem (Gene Chem, China). The GV230-VEGFA overexpression vector was constructed by deleting the *EphA2* gene from the GV230-EphA2 vector. The empty GV230 vector was used as a negative control.

### RNA-seq analysis

Isolated total RNA was sent to Shanghai Personal Biotechnology Co., Ltd. (Shanghai, China) for transcriptome sequencing. A library was prepared using the Illumina mRNA-Seq sample preparation kit and subjected to paired-end sequencing. Differentially expressed genes (DEGs) were defined as those with an absolute fold-change in expression >1.5 or <0.5. DAVID software was used to annotate GO functions of DEGs, including their cell components, biological processes, and molecular functions.

### m6A RNA immunoprecipitation-qPCR

m6A RNA immunoprecipitation (MeRIP)-qPCR assays were used to detect m6A modifications of individual genes. First, total RNA was extracted using RNAiso Plus (TaKaRa) and poly(A) + RNA was further purified using an mRNA Isolation System (Sigma, Missouri, USA). Subsequently, MeRIP was performed using a Magna MeRIP m6A kit (Millipore, Massachusetts, USA), according to the instructions and RIP enrichment determined by qPCR. m6A enrichment was determined in each sample by normalization to the input sample. The primers used for qPCR are listed in Supplementary Table S1.

### m6A mutation and luciferase reporter assays

EphA2-3ʹUTR and VEGFA-3ʹUTR fragments containing wild-type and mutant m6A motifs, were directly synthesized by GENEWIZ (Suzhou, China) and then inserted into the luciferase reporter vector, pMIR-REPORT. For dual-luciferase reporter assays, 100 ng wild-type or mutant EphA2 or VEGFA fragments, 200 ng shMETTL3/pcDNA3.1-FTO, and 20 ng pRL-TK (Renilla luciferase control reporter vector) were co-transfected into HEK-293T cells in 24-well plates. Forty-eight hours after transfection, the cells were lysed using passive lysis buffer and harvested. Luciferase activity was assayed using a Dual-Luciferase Reporter Assay System (Promega, Wisconsin, USA), according to the manufacturer’s instructions. The ratio of relative luciferase activity was normalized to that of Renilla luciferase activity. Each group was analyzed in triplicate.

### siRNA

siRNAs against IGF2BPs were designed using siDirect version 2.0 and DSIR and synthesized by Gene Pharma (Suzhou, China). Cells were transfected with siRNA using Lipofectamine 3000 (Invitrogen) at a final concentration of 20 nM in 24-well plates. After incubation for 48 h, cells were harvested for qRT-PCR and western blot analysis. The sequences of siIGF2BPs are listed in Supplementary (Supplementary Table S1).

### RNA stability

HCT116 wild-type and METTL3-knockdown cells were seeded in 12-well plates and treated with 5 μg/ml actinomycin D (Sigma, USA) at 0, 3, and 6 h. Total RNA was then isolated using RNAiso Plus (TaKaRa) and analyzed by qPCR. The mRNA degradation rate was estimated according to published protocols [22].

### Cell proliferation, invasion, migration, and colony formation

For cell proliferation assay, cells were seeded in 96-well plates and incubated overnight. Then, cell viability was measured by adding MTT for 0, 24, 48, and 72 h, and absorbance values at OD450 measured using a microplate reader (Bio-Rad, USA). Each group was analyzed in triplicate.

For analysis of cell invasion and migration, 1 × 10^6^ cells were seeded in the upper chambers of Transwell plates (Corning, NewYork, USA) coated with or without Matrigel (BD Biosciences, New Jersey, USA). After incubation for 48 h, cells were fixed in 4% paraformaldehyde for 20 min and stained with 0.1% crystal violet for 30 min. Migrating and invading cells were observed under a microscope, and cells from three fields counted using Image J software.

Cells (500 per well) were seeded into 6-well plates and maintained in DMEM containing 10% FBS for 2 weeks. Colonies were fixed and stained with 0.1% crystal violet for 30 min, then imaged and counted for statistical analysis.

### In vitro tube formation assay

Tubular structure formation was assessed using a Matrigel tube formation assay. Briefly, a total of 100 μL growth factor-reduced Matrigel (BD Biosciences, New Jersey, USA) was added to each well of a precooled 48-well plate and solidified at 37 °C overnight. Cells were suspended in DMEM and seeded on a 48-well plate embedded with Matrigel. After incubation for 48 h, the extent of tubular structure formation in each group was determined by examination under an Olympus inverted microscope (Tokyo, Japan).

### Mouse xenograft model

Nude mice were purchased from Charles River and raised under specific pathogen-free conditions. A total of 8 × 10^6^ wild-type (WT) or METTL3-knockdown cells were injected into the dorsal flanks of 6-week-old nude mice. Seven mice were randomly selected to calculate the volume according to the following formula: V = (width^2^ × length)/2. Mice were euthanized three weeks after injection and tumors removed, weighed, fixed, and embedded for immunohistochemical analysis. Experiments exclude conscious or subconscious personal subjectivity and preference of operators and participants. Animal studies were performed in accordance with the institutional ethics guidelines for animal experiments, and approved by the Institutional Animal Care and Use Committee or Animal Experimental Ethics Committee of Harbin Institute of Technology (HIT).

### Immunohistochemical staining

Sections were deparaffinized in xylene and hydrated with gradient ethanol (100%, 90%, 75%, and 50%, for 5 min each). Sections were then microwaved-heated in sodium citrate buffer for antigen retrieval. After blocking in 2% BSA/PBS, sections were incubated with anti-METTL3/CD31 (Abcam, 1:200 dilution) or anti-EphA2/VEGFA (CST, 1:100 dilution) at 4 °C overnight. Next, HRP-conjugated rabbit secondary antibody was added to the sections and incubated for 60 min at room temperature. Finally, signals were developed using a DAB kit (ZSGB-BIO, China), according to the manufacturer’s instructions, and images obtained using an Olympus inverted microscope (Japan). Hematoxylin was used for counterstaining.

### Statistical analysis

All data in this study were generated from at least three independent replicate experiments. Data are presented as the mean ± standard deviation (SD). Statistical significance was tested using the two-tailed Student’s *t* test and is indicated by *p* values; *p < 0.05 and **p < 0.01 were considered to indicate significance. Statistical analyses were performed using GraphPad Prism software.

## Supplementary information

Figure S1

Figure S2

Table 1

Table 2

Original Data File

check list

## Data Availability

The raw sequencing data have been deposited in the Gene Expression Omnibus database under the accession number. All the other data generated in this study are included in the article and the additional files.
