# Supplementary material for: m6A methylated EphA2 and VEGFA through IGF2BP2/3 regulation promotes vasculogenic mimicry in colorectal cancer via PI3K/AKT and ERK1/2 signaling
Source: Cell Death Dis. 2022 May 21;13(5):483. doi: 10.1038/s41419-022-04950-2 (PMC9122982; doi:10.1038/s41419-022-04950-2)

**Figure S1,** related to Fig.3. Identification of METTL3 targets via MeRIP-qPCR

a, The m6A modification site of EphA2 predicted by SRAMP website tools, and primers designed for MeRIP-qPCR assay. b, The m6A modification site of VEGFA predicted by SRAMP website tools, and primers designed for MeRIP-qPCR assay.

a.


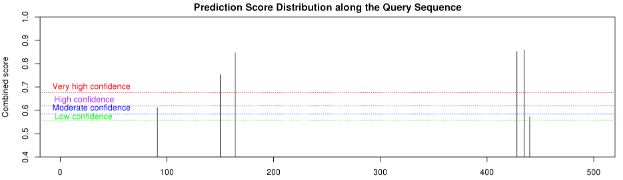


b.
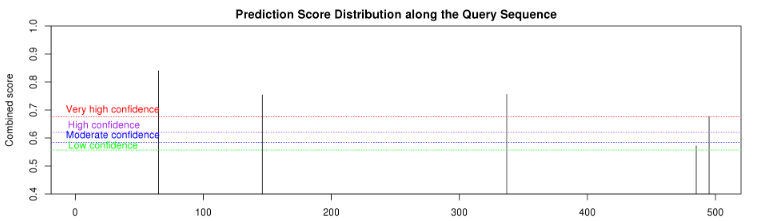

Supplement: Supplementary file 1 — Figure S1 [file 41419_2022_4950_MOESM1_ESM.docx]
