# Supplementary material for: m6A methylated EphA2 and VEGFA through IGF2BP2/3 regulation promotes vasculogenic mimicry in colorectal cancer via PI3K/AKT and ERK1/2 signaling
Source: Cell Death Dis. 2022 May 21;13(5):483. doi: 10.1038/s41419-022-04950-2 (PMC9122982; doi:10.1038/s41419-022-04950-2)

**Figure S2** related to Fig.4: The efficiency of IGF2BP1 knockdown and EphA2 and VEGFA was measured by qRT-PCR. a, The expression of IGF2BP1 was detected by qRT-PCR after knocking down IGF2BP1. b-c, Real-time PCR assay of EphA2 and VEGFA expression in HCT116 cells after depletion of m6A readers (IGF2BP1). p values were calculated using the t-test. *p < 0.05, **p < 0.01, ***p < 0.001.


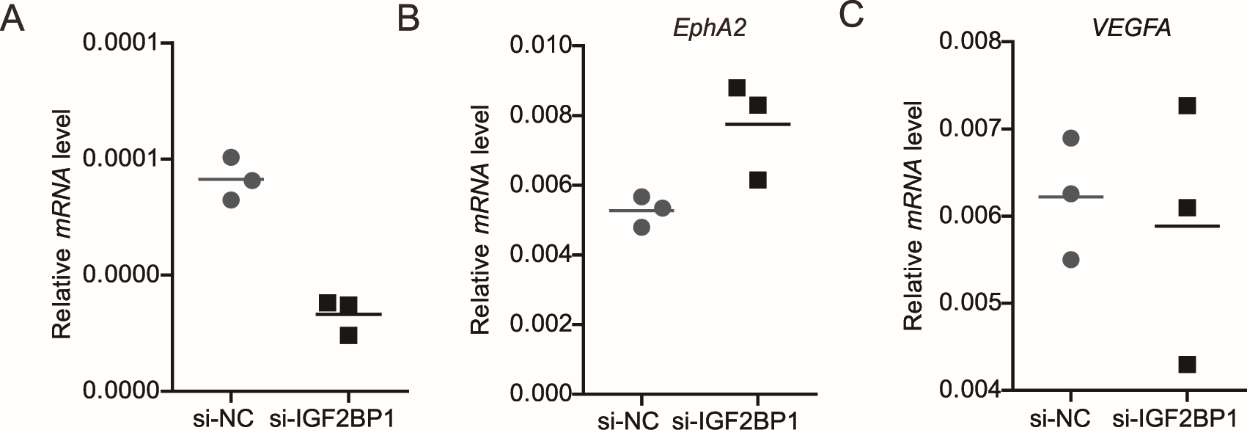

Supplement: Supplementary file 2 — Figure S2 [file 41419_2022_4950_MOESM2_ESM.docx]
