# Supplementary material for: m6A methylated EphA2 and VEGFA through IGF2BP2/3 regulation promotes vasculogenic mimicry in colorectal cancer via PI3K/AKT and ERK1/2 signaling
Source: Cell Death Dis. 2022 May 21;13(5):483. doi: 10.1038/s41419-022-04950-2 (PMC9122982; doi:10.1038/s41419-022-04950-2)
Supplement: Supplementary file 3 — Table 1 [file 41419_2022_4950_MOESM3_ESM.docx]

**Table1 Primer Sequences**

| **Primer name** | **Primer sequence** |  |
| --- | --- | --- |
| sgRNA-METTL3 | GAGAGGCTGCAGCGGAGG |  |
| shRNA-METTL3 | GCTAAACCTGAAGAGTGATAT |  |
| METTL3-F | CTCTGCGGGAGAGGCTGCAG | qRT-PCR-Primers |
| METTL3-R | GAGAAGAAGTTGCTACACCAC |  |
| EphA2-F | TGCCAGTGTCAGCATCAACCAG |  |
| EphA2-R | AGTCTCCCTTCTTGCGGTAAGTG |  |
| FTO-F | GGCAGTGTACAGTTATAGCTGTG |  |
| FTO-R | CCAAACCAGGTGTCTCTATGTC |  |
| EphA2-F | TGCCAGTGTCAGCATCAACCAG |  |
| EphA2-R | AGTCTCCCTTCTTGCGGTAAGTG |  |
| VEGFA-F | TCCGCAGACGTGTAAATGTTCC |  |
| VEGFA-R | GCGTGGTTTCTGTATCGATCGT |  |
| EGR1-F | AGCAGCACCTTCAACCCTCAGG |  |
| EGR1-R | GCTGCAGCCAGCTTGTTCCT |  |
| TRIB3-F | GCTTTGTCTTCGCTGACCGTGA |  |
| TRIB3-R | CTGAGTATCTCAGGTCCCACGT |  |
| DDIT4-F | AGGACGCACTTGTCTTAGCAG |  |
| DDIT4-R | GCAAGGACGAGGGCGAAGAG |  |
| CLDN1-F | GATTTACTCCTATGCCGGCGACAA |  |
| CLDN1-R | CATCTTCTGCACCTCATCGTCT |  |
| JAG1-F | GACTCATCAGCCGTGTCTCA |  |
| JAG1-R | TGGGGAACACTCACACTCAA |  |
| CEBPG-F | CCTGCACTTTACTGTTGAACGTAG |  |
| CEBPG-R | TCTCCCTTGCCAACACAGAAC |  |
| PDE4D-F | GCATGGTATGGCAATAGAATCG |  |
| PDE4D-R | GCCTTGGTGTTGGGTAAGAGG |  |
| ZFP36L1-F | CCCCGACCTTGGACAACTCA |  |
| ZFP36L1-R | AGCACCAAAATCAAGTCTCTCC |  |
| IGF2BP1-F | AACGACCAGGTCATCGTGAA |  |
| IGF2BP1-R | TCTCGATTTCTGCCCGTTGT |  |
| IGF2BP2-F | ACCAGTGCAGAAGTCATCGT |  |
| IGF2BP2-R | GGAAGGGCTACATTCATCCGTT |  |
| IGF2BP3-F | GCTCATATCAGAGTGCCATCCT |  |
| IGF2BP3-R | TTTGGCATCTGCCTCTGTGGT |  |
| GAPDH-F | AGAAGGCTGGGGCTCATTTG |  |
| GAPDH-R | AGGGGCCATCCACAGTCTTC |  |
| OE-FTO-F | AAACTCGAGATGAAGCGCACCCCGACTGC |  |
| OE-FTO-R | AAAGCGGCCGCTAGGGTTTTGCTTCCAGAG |  |
| OE-VEGFA-F | GGACTCGAGCTGACGGACAGACAGACAGC |  |
| OE-VEGFA-R | GTTGGTACCTCACCGCCTCGGCTTGTCAC |  |
| EphA2-M6A-F1 | CCTGCAACTTCCGCTGAGG | M6A-RIP-qPCR- primers |
| EphA2-M6A-R1 | TGCTCAGCTGTGTGCGTCTC |  |
| EphA2-M6A-F2 | GTAAACAGGGTACCTCAAGC |  |
| EphA2-M6A-R2 | AAGGCACTAGAGGGACAGG |  |
| EphA2-RIP-N-F | TGCCAGTGTCAGCATCAACCAG |  |
| EphA2-RIP-N-R | AGTCTCCCTTCTTGCGGTAAGTG |  |
| VEGFA-M6A-F1 | TCAGGGTTTCGGGAACCAG |  |
| VEGFA-M6A-R1 | TCGATGGTGATGGTGTGGTG |  |
| VEGFA-M6A-F2 | CCACCACACCATCACCATCG |  |
| VEGFA-M6A-R2 | CGGACCCAAAGTGCTCTGC |  |
| VEGFA-M6A-F3 | ACGGTCCCTCTTGGAATTGG |  |
| VEGFA-M6A-R3 | TGTATGTGGGTGGGTGTGTC |  |
| VEGFA-M6A-F4 | CAGTGCTAATGTTATTGGTGTC |  |
| VEGFA-M6A-F4 | CCTGTCAGGATCTGAGTGG |  |
| VEGFA-RIP-N-F | TGTCTTGGGTGCATTGGAG |  |
| VEGFA-RIP-N-R | CCATGAACTTCACCACTTCG |  |
| EphA2-REPORT-F | CACACTAGTTCCCTGCTGTGCCATGCTG | Dual-Luciferase reporter Primers |
| EphA2-REPORT-R | GCCAAGCTTCCAGGAAAGCAAGGGTTTGG |  |
| VEGFA-REPORT-F | GAGACTAGTAGGGTTTCGGGAACCAGATC |  |
| VEGFA-REPORT-R | CTCAAGCTTCCCAACTCAAGTCCACAGC |  |
| si- IGF2BP1-sense | GGAAAUAAUGAAGAAAGUUCGTT | siRNA sequences |
| si- IGF2BP1-antisense | CGAACUUUCUUCAUUAUUUCCTT |  |
| si- IGF2BP2-sense | GAGAUAGAGAUUAUGAAGATT |  |
| si- IGF2BP2-antisense | UCUUCAUAAUCUCUAUCUCTT |  |
| si- IGF2BP3-sense | GGUGAAUGAACUUCAGAAUTT |  |
| si- IGF2BP3-antisense | AUUCUGAAGUUCAUUCACCTT |  |
| Myco-F | YGCCTGRGTAGTAYRYWCGC | mycoplasma |
| Myco-R | GCGGTGTGTACAARMCCCGA | detection primer |
