# Supplementary material for: m6A methylated EphA2 and VEGFA through IGF2BP2/3 regulation promotes vasculogenic mimicry in colorectal cancer via PI3K/AKT and ERK1/2 signaling
Source: Cell Death Dis. 2022 May 21;13(5):483. doi: 10.1038/s41419-022-04950-2 (PMC9122982; doi:10.1038/s41419-022-04950-2)
Supplement: Supplementary file 4 — Table 2 [file 41419_2022_4950_MOESM4_ESM.docx]

**Table S2.** The significantly down-regulated 10 candidates m6a sites were predicted via SRAMP database and RMBase2.0/BERMP.

| SRAMP | Motif score | | RMBase  2.0 | | Motif score (SP) | | BERMP | Motif score |
| --- | --- | --- | --- | --- | --- | --- | --- | --- |
| EPHA2 | 0.826 | DDIT4 | | 419.59(82) | | VEGFA | | 0.2995 |
| VEGFA | 0.819 | VEGFA | | 419.59(72) | | TRIB3 | | 0.29133 |
| PDE4D | 0.807 | EPHA2 | | 419.59(46) | | EPHA2 | | 0.24416 |
| TRIB3 | 0.783 | PDE4D | | 419.59(43) | | DDIT4 | | 0.19796 |
| DDIT4 | 0.749 | ZFP36L1 | | 419.59 | | CLDN1 | | 0.17834 |
| CLDN1 | 0.711 | TRIB3 | | 371.87 | | PDE4D | | 0.17617 |
| ZFP36L1 | 0.681 | EGR1 | | 371.87 | | EGR1 | | 0.1609 |
| CEBPG | 0.694 | CLDN1 | | 371.87 | |  | |  |
|  |  | CEBPG | | 349.63 | |  | |  |
| Annotation：Motif score :SRAMP (0-1) / RMBase2.0（0-500）SP: support number/ BERMP 0-1) | | | | | | | | |
