# Supplementary material for: m6A methylated EphA2 and VEGFA through IGF2BP2/3 regulation promotes vasculogenic mimicry in colorectal cancer via PI3K/AKT and ERK1/2 signaling
Source: Cell Death Dis. 2022 May 21;13(5):483. doi: 10.1038/s41419-022-04950-2 (PMC9122982; doi:10.1038/s41419-022-04950-2)
Supplement: Supplementary file 5 — Original Data File [file 41419_2022_4950_MOESM5_ESM.docx]

**m6A methylated EphA2 and VEGFA through IGF2BP2/3 regulation promotes vasculogenic mimicry in colorectal cancer via PI3K/AKT and ERK1/2 signaling**

XinLiu^1^, Hongjuan He^1^, Fengwei Zhang^1^, Xin Hu^1^, Fanqi Bi ^2^, Kai Li^1^, Haoran Yu^1^, Yue Zhao^3^, Xiangqi Teng^1^, Jiaqi Li^1^, Lihong Wang^1^, Yan Zhang^2^* & QiongWu^1^*^[[1]](#footnote-1)^

1School of Life Science and Technology, State Key Laboratory of Urban Water Resource and Environment, Harbin Institute of Technology, Harbin 150001, Heilongjiang, China.

2 School of Life Science and Technology, Computational Biology Research Center, Harbin Institute of Technology, Harbin 150001, Heilongjiang, China

3 Department of Urology, Xiang'an Hospital of Xiamen University, Xiamen, 361000, Fu Jian, China

Supplementary Material

(Western Blots)


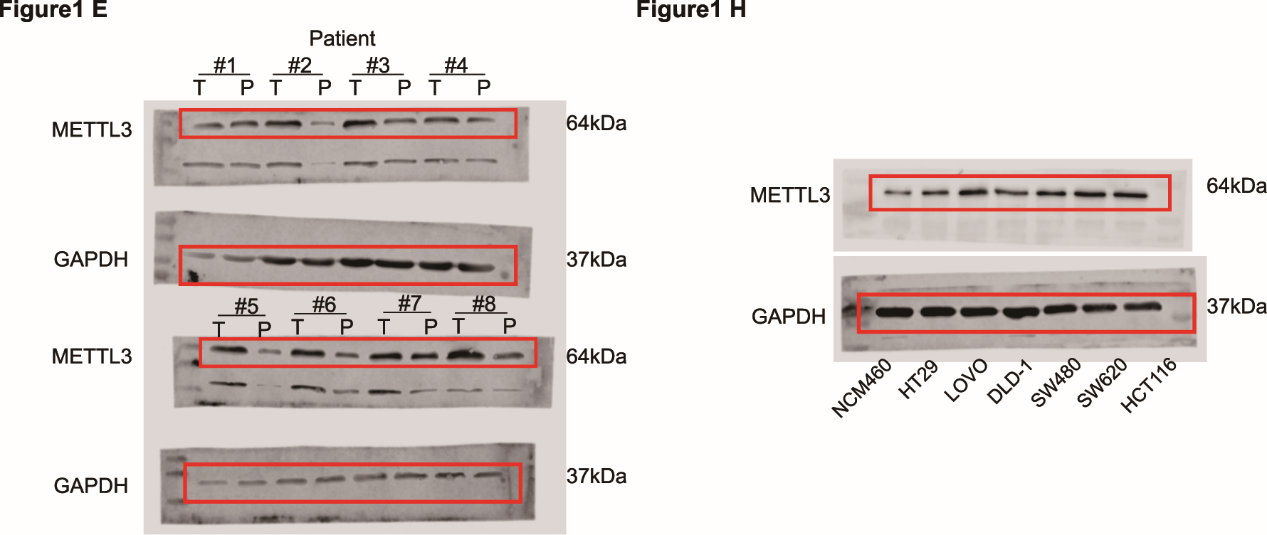


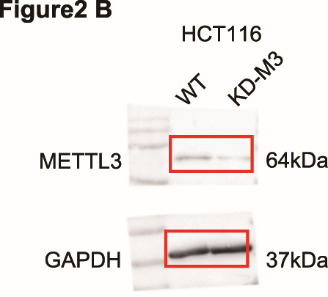


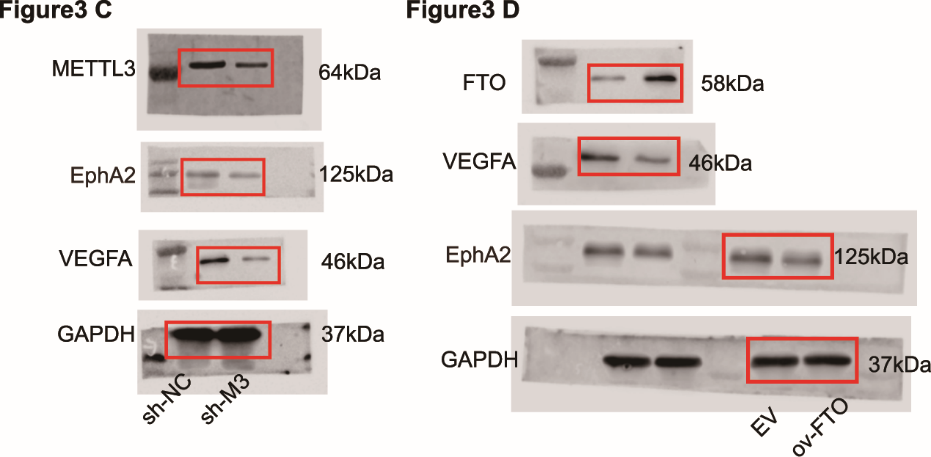


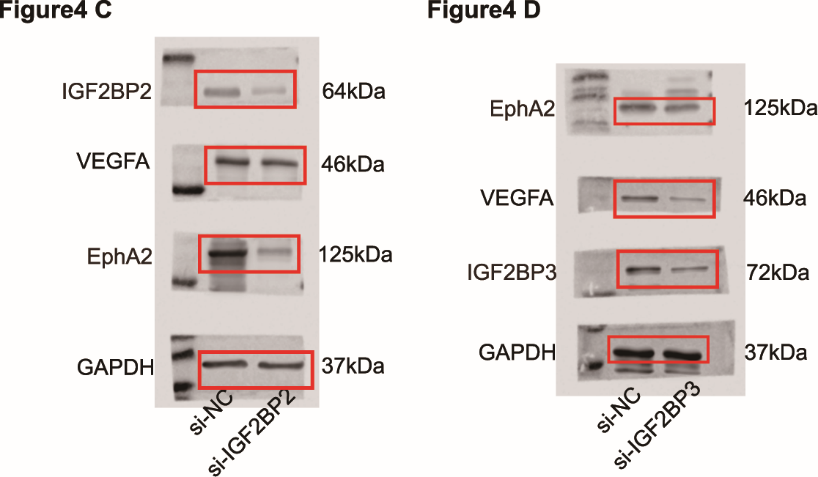


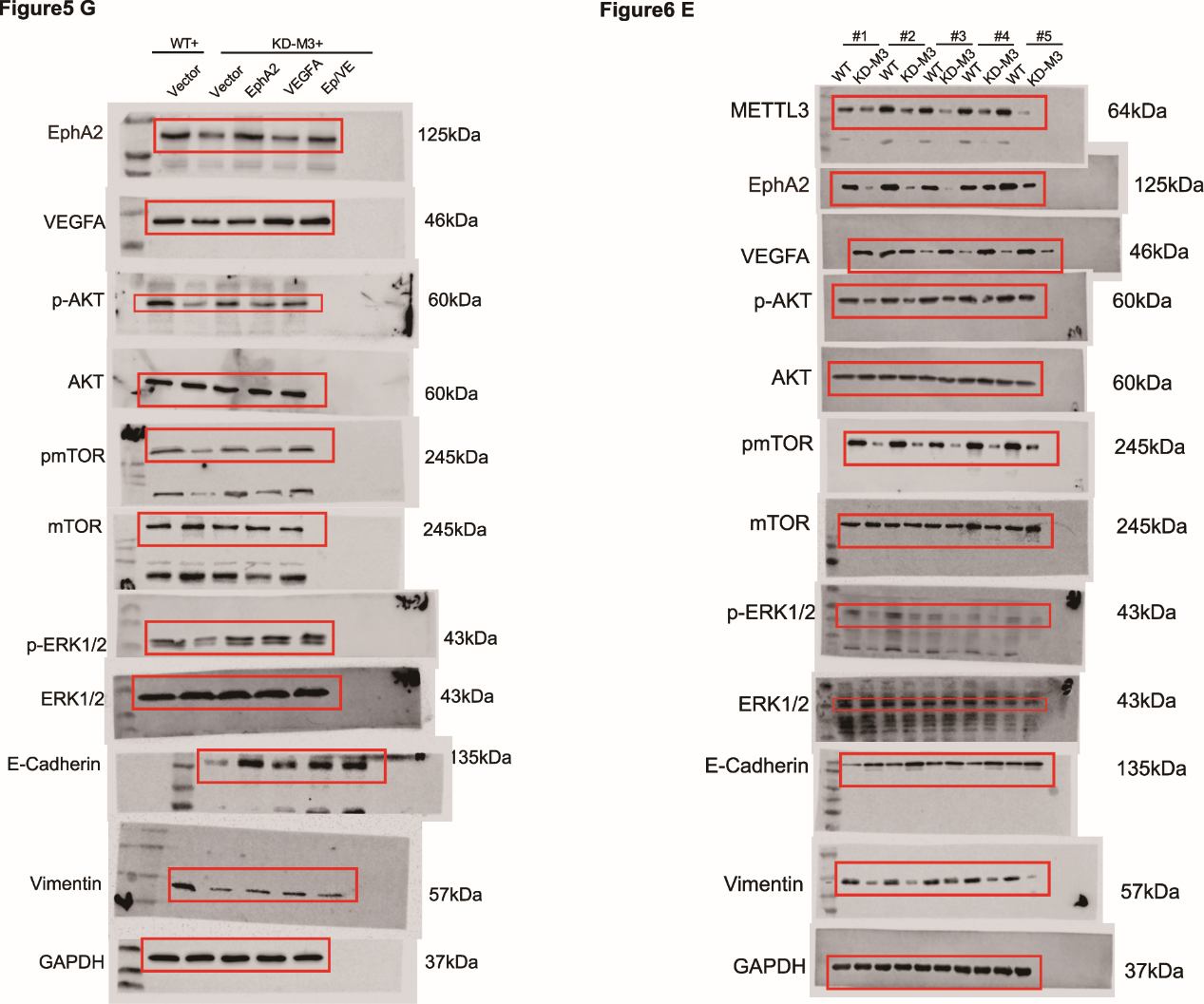


Supplementary Material

(Dot Blots)


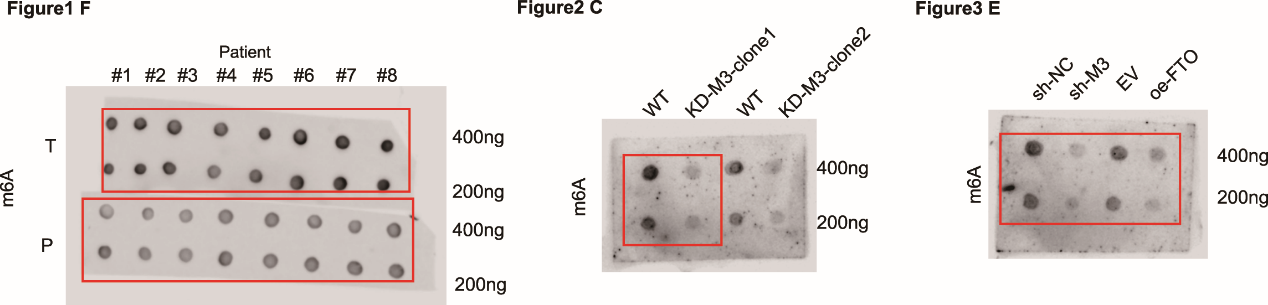


1. **Correspondence:** Qiong Wu (kigo@hit.edu.cn) & Yan Zhang (hangtyo@hit.edu.cn); [↑](#footnote-ref-1)
